# Self-Medication-Related Behaviors and Poland’s COVID-19 Lockdown

**DOI:** 10.3390/ijerph17228344

**Published:** 2020-11-11

**Authors:** Marta Makowska, Rafał Boguszewski, Michał Nowakowski, Monika Podkowińska

**Affiliations:** 1 Institute of Sociological Sciences and Pedagogy, Warsaw University of Life Sciences, Nowoursynownska 166 St. 02-787 Warsaw, Poland; marta_makowska@sggw.edu.pl (M.M.); monika_podkowinska@sggw.edu.pl (M.P.); 2 Department of Social Health Problems, Maria Curie-Skłodowska University in Lublin, Plac Marii Curie-Skłodowskiej 5, 20-031 Lublin, Poland; m.nowakowski@poczta.umcs.lublin.pl

**Keywords:** self-medication, lockdown, COVID-19, Poland

## Abstract

(1) Background: The SARS-CoV-2 pandemic has changed the functioning of Polish health systems. Telemedicine has been developed and access to prescription drugs (Rx) has been facilitated. This study examined whether these changes and the imposition of a three-month lockdown caused Polish people to engage in more self-medication-related behaviors. (2) Method: After the fourth (final) stage of defrosting the Polish economy, an online survey of a quota sample of 1013 Polish respondents was conducted. (3) Results: Almost half of the respondents (45.6%) indicated that they had engaged in at least one behavior associated with inappropriate self-medication during the lockdown (e.g., 16.6% took medication as a precaution, and 16.8% took an Rx formulation without consultation). Some of these people had never engaged in such behaviors prior to the lockdown. Linear regression showed that higher values of a composite (“lockdown”) index of self-medication-related behaviors occurring during lockdown were predicted by greater religiosity and the presence of children in a household. Also, independent samples *t*-tests showed that people who were afraid for their financial future and people who feared for their health obtained higher lockdown index scores than people not having such worries. (4) Conclusions: Self-medication-related behaviors were more common among Poles before lockdown than during the lockdown (which is unsurprising given that the lengths of the periods compared were hugely different), worryingly, many people exhibited such behaviors for the first time during the lockdown.

## 1. Introduction

The SARS-CoV-2 pandemic has changed the way people live in most countries in the world. From December 2019—when the virus was first detected—to July 2020, 13.5 million infections were diagnosed in 188 regions of different countries [1]. In response to the pandemic, some countries (including Poland) have introduced rules mandating social distancing, the identification and isolation of infected people, and the quarantining of people who have recently been in physical proximity to them. Societies have also experienced changes in rules regarding access to medical services and products, and scientific literature has begun to appear which suggests that the pandemic is influencing self-medication behaviors. Such behaviors seem to be mainly attributable to media suggestions that people may repurpose certain medications with a longstanding presence in the market as effective COVID-19 treatments or preventives [2,3]. However, it has been emphasized that such behaviors may have dangerous consequences in the form of side effects, fatalities, or shortages of these drugs for patients who are usually treated with them [3].

According to the World Health Organization (WHO, Geneva, Switzerland; 2000), self-medication is an important element of self-care, and is defined as the taking of medications to heal self-diagnosed problems or the self-administration of medications prescribed by a doctor in the case of chronic diseases, recurring diseases, or symptoms [4]. Self-medication is also defined as taking medication on one’s own initiative or on the initiative of someone who is not medically qualified [5,6].

The literature distinguishes between so-called responsible self-medication and inappropriate self-medication. The former refers to use of over-the-counter (OTC) drugs in accordance with package instructions or use of prescribed (Rx) medications approved by medical professionals. In addition to making more efficient use of health budgets and the time of doctors and pharmacists, responsible self-medication allows greater empowerment of patients by involving them in their treatment [4,7,8]. All of these constitute major advantages at a time when healthcare systems are inundated with COVID-19 cases.

Inappropriate self-medication is a particularly apt term to use where people take medication irresponsibly [9]. This involves taking prescription drugs without a prescription, using old drugs prescribed for other ailments, sharing medications with friends/family, and using out-of-date drugs [10]. Although the literature describes the inappropriate use of OTC drugs [11,12,13,14], the inappropriate use of Rx drugs has received more attention [13]. An issue of particular interest to researchers has been self-medication with antibiotics [6,15], this being recognized as one of the most common causes of antibiotic resistance [6], and there is evidence that patients exert pressure on doctors to (inappropriately) prescribe antibiotics for viral infections [16,17]. Research has also focused on the use of Rx drugs for the enhancement of physical performance (both athletic and sexual) and cognitive enhancement [18,19,20]. This phenomenon is of interest not only to public health experts, but also to sociologists who have developed the concept of pharmaceuticalization [21,22].

Inappropriate self-medication carries many dangers, such as: incorrect self-diagnosis and inappropriate choice of therapy; delaying the seeking of medical advice; various side effects of wrongly taken medications; the taking of multiple medications—which may have unforeseen interactions and/or produce interactions with certain foods; the taking of incorrect drug dosages; incorrect administration; risk of addiction; storage of medication in inappropriate conditions [7]. Mallhi et al. stated that even if only 0.1% of instances of inappropriate self-medication were to result in complications, this would amount to thousands of cases, with which a healthcare system engaged in fighting the COVID-19 pandemic would find it difficult to cope with [3].

Rather et al. [6] point out that self-medication can occur because making an appointment with a doctor can be inconvenient, and people can delay this until a disease becomes serious. This is likely to be particularly true given the restrictions placed on access to medical care during the pandemic: such restrictions are likely to have further increased the propensity to self-medicate. Also, medical systems in some countries have been so overburdened with COVID-19 cases that patients with other conditions have not received adequate care [23], and physical visits to a GP’s premises or a hospital may be seen as highly dangerous by some people, with doctors and nurses being seen as potential carriers of the virus [24,25]: both of these factors may increase the risk of people’s propensity to self-medicate.

A rapid increase in self-medication among Poles during the 1990s is likely to have been caused by political change. The emergence of the free market led to the appearance of many new medications in Poland, and the availability of drugs for patients improved significantly [26]. These developments were also supported by changes in pharmaceutical laws in 1994, which made it possible to advertise OTC drugs [27]. In 1996, TNS OBOP conducted research on Poles’ behavior concerning diseases and ailments. This showed that as many as 59% of Poles used various forms of self-medication—11% using safe OTC drugs and 48% using home remedies [27]. Also, a 2010 Public Opinion Research Centre study found that 21% of people admitted to misusing OTC drugs [28], and a 2016 study indicated that this had risen to 28% [29]. A final observation to note here, is that in 2006 Poland participated in an international study on self-medication with antimicrobial drugs, which showed that 33 people per 1.000 inhabitants took such medication without consulting a physician. Lower levels of self-medication were found in 12 of the 19 countries studied [30].

With respect to the COVID-19 pandemic, the Polish situation during the spring 2020 was not as bad as in other European countries such as Italy and Belgium, but it was worse than in countries such as its neighbor Slovakia. On June 8, 2020, 27,160 infections and 1166 deaths had been identified [31]. To date, the Polish healthcare system has not become overloaded, but the pandemic has accelerated digitization and many new procedures have been developed [32]. Many planned surgical procedures were canceled during a governmentally mandated lockdown [33], new safety procedures have made the access to universal healthcare difficult, telephone medical advice has become common, and in most cases, this has replaced physical visits. In this unprecedented context, and its previously discussed potential implications for self-medication behaviors, we thought it is important to examine the impact of the pandemic and the associated lockdown on Polish people’s self-medication tendencies. The study sought to answer the following research questions: (1) How did the lockdown associated with the pandemic influence Poles’ self-medication-related behaviors? (2) What are the characteristics of Poles whose self-medication-related behaviors exhibited the most change during the lockdown period? 

## 2. Materials and Methods

An online survey using one of the largest Internet panels in Poland (the SW Research Panel) was conducted between 8 June and 15 June 2020. The survey was carried out just after implementation of the last (fourth) stage of the Polish government’s defrosting process on 6 June, which allowed Poles to return to a “new normality” [34]. Respondents were asked about their experiences during their three-month lockdown, which ran from 11 March to 6 June. The restrictions during this period influenced many spheres of Polish life. For example, educational institutions were closed, many companies recommended remote working, cinemas and theaters were closed, and there were restrictions on religious practices. 

A purposely developed structured questionnaire was used. This consisted of 64 questions, including 2 multiple-choice questions, 46 statements involving 5-point Likert scale responses, and 16 socio-demographic questions. The questionnaire was created by the authors following the procedures outlined by Malhotra [35], and referred only to the Polish socio-cultural context given that Poland was the only country involved in the study. The content validity of the questionnaire was ensured via consultations with experts and pilot testing. Extensive validation was not possible because of the time critical nature of the research, but the questionnaire’s novelty, and the fact that its use was limited to a single nation, meant that no validation issues relating to translation arose and no adaptations to the specificity of the culture were necessary. The questionnaire and resulting database are available on figshare: https://figshare.com/articles/dataset/Poland_-_Covid_-19_Second_Wave/13071875 (Supplementary Materials). Ethics committee consent was not obtained, as Polish regulations do not require this for online surveys. Based on their socio-demographic characteristics, all participants received an invitation to take part in the study from SW Research and could complete the questionnaire only once. All participation was voluntary, and participants received a small amount of remuneration in a form they chose from the SW Research Panel’s rewards pool. The Research Panel’s processes ensured that participants consent to their data being used under the provisions of the EU’s General Data Protection Regulation. After data are provided to researchers, it is necessary to encode databases so that it is impossible to identify individual research participants. Thus, while participants were anonymous to the researchers after data were encoded, they were not anonymous to SW Research.

In Poland, around 20 million adults have access to the Internet. Assuming an alpha of 0.05, desired power of 0.80, a (small) effect size of d = 0.2, and an allocation ratio of 0.25 for *t*-tests, G*Power 3.1 [36] suggested a sample of 968 people. But, since the actual allocation ratios that would occur for different analyses were unknown, we erred on the side of caution and aimed to gather data from 1000 respondents. So, a quota sample of 1013 people was obtained, the sample being representative of the Polish adult population in terms of gender (2 groups), age (5 groups), population size of place of residence (4 groups), province (16 groups), and education (2 groups: higher and other). Appendix A contains distributions of respondents’ socio-demographic characteristics. Respondents took a mean time of 9 min, 35 s, and a median time of 8 min, 6 s to complete the questionnaire. These statistics exclude participants who completed the questionnaire either too quickly (less than 3 min) or too slowly (more than an hour); data for 24 people were excluded on these grounds.

Data were analyzed using IBM SPSS Statistics (v. 26). A number of analytic techniques were used, starting with frequency distribution and crosstabulation analyses. Two overall self-medication indices were constructed to examine the multidimensional relationships. These indices were created from responses to six statements describing behaviors connected with self-medication across two different periods of time. A general index covered respondents’ whole lives (including lockdown), and a lockdown index covered only the three-month lockdown period. Two bivariate correlation matrices (one for people’s whole lives both before the lockdown period and during the lockdown period, and one for the lockdown period only; see Appendix B) were obtained to ascertain whether responses to statements used to derive each index were correlated. The correlations were mostly in the small to medium range in terms of Cohen’s benchmarks, and none were large enough to warrant a concern that any of the statements were measuring exactly the same thing conceptually, this providing a basis to take all of the statements into account in creating the indices. Respondents could achieve a maximum of 6 points, 1 point for each declaration that a given behavior had taken place. Cronbach’s alpha coefficients were computed to assess the reliability of both indices, and these were found to be reasonable (α = 0.77 for the general index, and α = 0.72 for the lockdown index). To investigate whether independent relationships existed between various factors and values of the indices, two linear regression analyses were performed, one with each index as a dependent variable. The independent variables in each analysis were: gender, age (grouped into ranges), population size of place of residence, level of education, participation in religious practices, life satisfaction, self-assessed health, and the presence/absence of children under 18 years-old in the household. Independent samples *t*-tests were used to examine the differences in lockdown index values for groups differing in acceptance of various individual statements relating to the pandemic. 

## 3. Results

Initially, we examined the frequencies of different types of self-medication before and during the lockdown (see Table 1). It should be emphasized that not all responses necessarily imply inappropriate self-medication: refraining from consulting a doctor despite experiencing worrying symptoms and/or buying prescription medications before they are needed do not necessarily indicate the taking of medication, but not seeing a doctor when one feels unwell, and having ready access to drugs, will both increase the probability of self-medication occurring.

For all time points aggregated, “Never” responses to individual questions concerning self-medication ranged from 59% to 80%. The most common type of behavior that respondents admitted to was sometimes refraining from consulting a doctor despite having worrying symptoms (almost 42%). Also, a little less than 40% of respondents had taken prescription drugs without medical consultation and had bought such drugs before they were needed.

In the three-month period from the announcement of the lockdown (March 11) to the end of data collection (15 June), some Poles’ self-medication behaviors changed. Table 1 shows that, according to respondents’ declarations, 15.5% of people who had never previously bought prescription drugs before they were needed, did so, and as many as 12.1% of respondents with worrying symptoms who previously would have seen a doctor, did not do so. Also, every ninth respondent (11.3%), who did not normally take medication on a precautionary basis did so, and a very similar number (10.8%) took medication to enhance physical/cognitive performance for the first time. Finally, 10.2% of people took prescription drugs without a medical consultation when they had not previously done this, and every thirteenth respondent (7.0%), who had not previously done so talked a doctor into prescribing them drugs. 

Crosstabulations and chi-square tests were performed to identify which socio-demographic characteristics differentiated between people engaging in self-medication-related behaviors only before the lockdown and people engaging in these behaviors both before and during the lockdown. While most of the tests were nonsignificant, a few significant associations were identified. Significantly more people than expected with two children under 18 in their household had taken prescription medication without consulting a doctor both before and during the lockdown. Also, people who had made precautionary purchases of prescription medications both before and during the lockdown more often than would be expected: (a) did not know if they were satisfied or dissatisfied with their lives; (b) assessed their health as poor or very poor, and; (c) had participated in religious rituals only once every few years. Finally, fewer people than expected who had taken medication as a precaution against becoming ill both before and during the lockdown were: (a) in the 60+ year-old age range and (b) dissatisfied with life. Also, less males than expected had bought prescription medicines on a precautionary basis before the lockdown only (and more than expected both before and during the lockdown), and the reverse occurred for females (see Appendix C for details). 

**Figure 1:**
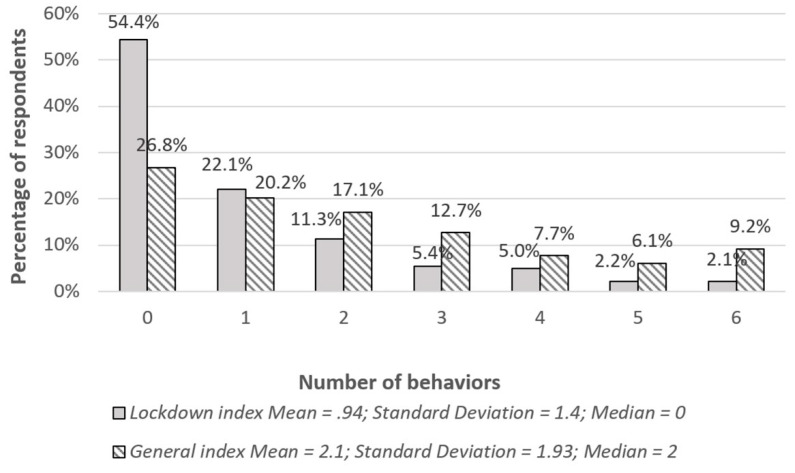
Percentages for the general and lockdown indices.

Two indices were created based on the items in Table 1. The first, termed a general index, concerned the whole period of respondents’ lives (including the lockdown period), while the second, termed a lockdown index, applied only to behaviors during the three-month-lockdown period. Detailed information about the indices is presented in Figure 1.

To check if individual socio-demographic characteristics were independently predictive of values on each index, two linear regression analyses were performed (see Table 2). The model for the general index was significantly predictive, but it only explained a small amount of variability in the index (*R*^2^ = 0.058, *p* < 0.001). Controlling for the other predictor variables, the following variables were independently predictive: education (the higher the level of education, the greater the propensity to self-medicate), life satisfaction (the higher the life satisfaction, the greater the self-medication propensity), self-assessment of health (the worse the assessment, the greater the tendency to self-medicate), and having children under 18 in a household (people with children had a greater propensity to self-medicate than those without children). The model for the lockdown index was also significantly predictive, but again only a small amount of variability in the index was explained (*R*^2^ = 0.024, *p* < 0.001). When controlling the other variables, the only variables that were independently predictive were: frequency of religious practice (the greater the frequency, the higher the propensity to self-medicate), and having children under 18 in a household (people with children again having a higher propensity to self-medicate than those without children).

To identify the characteristics of people with a greater tendency to engage in self-medicating-related behaviors (as indicated by higher mean index values), independent samples *t*-tests and one-way between-subjects ANOVAs were conducted. People differing across gender and age (see Appendix A for details of age groupings) did not obtain significantly different values on either index. However, the results of the *t*-tests in Table 3 show that people who were dissatisfied with their lives obtained significantly higher mean values on the general index than those who were satisfied. Also, for both indices, people with children under 18 years old in their household obtained significantly higher mean values than those where this was not the case, and religious people (defined in terms of frequency of participation in religious practices) obtained significantly higher values than non-religious people. In all cases, effect sizes were small with respect to Cohen’s benchmarks for *d*.

An initial one-way between-subjects ANOVA showed significant main effects of level of education for both indices, with post hoc Bonferroni tests showing that the only significant differences between groups (*p* < 0.05) were those between people with a primary, lower secondary or vocational education and those with a secondary education. For both indices, people with the lower level of education showed a greater tendency to engage in the behaviors studied (see Table 3).

A further one-way ANOVA showed a significant main effect of the (population) size of people’s place of residence for the general index. Here, post-hoc Bonferroni tests showed that people from smaller towns (with up to 100,000 residents) had a significantly lower propensity to engage in the behaviors studied than those from the largest towns (over 500,000 residents). A final one-way ANOVA revealed a significant main effect of self-assessment of health for the general index. Post-hoc tests showed that people assessing their health as bad had a significantly higher propensity to engage in the behaviors considered than those rating it as good or very good. Also, people assessing their health as moderate had a higher propensity to exhibit the behaviors than those assessing their health as good or very good (see Table 4).

To obtain a better characterization of people who were more prone to engage in behaviors connected with self-medication during the lockdown period, independent samples *t*-tests were used to compare the lockdown indices of people answering “yes” vs. “no” to items assessing respondents’ opinions, behaviors, and knowledge regarding the pandemic (see Table 5). 

Compared to people who did not make such declarations, people who declared they feared for their own health, feared for the health and lives of their loved ones, declared worse mental well-being, and declared economic fears (of becoming financial broke and losing their job) had significantly higher values on the lockdown index. This was also true for people who made various attempts to avoid the hazard posed by the virus (e.g., by acquiring a large food supply to allow them to stay at home) compared to those who did not make such attempts. Also, respondents declaring that they would not be sure what to do if they observed coronavirus symptoms in themselves or other household members, and those who would not immediately contact the appropriate infectious disease hospital or sanitary department if they developed coronavirus symptoms were characterized by significantly higher lockdown indices. Finally, people agreeing with the controversial theory that pharmaceutical companies are responsible for releasing SARS-CoV-2, and that COVID-19 can be combated with a better diet or alternative medications rather than medical drugs, also had significantly higher lockdown indices than those disagreeing with such opinions. In all cases, effect sizes were small with respect to Cohen’s benchmarks for *d*.

## 4. Discussion

Examination of the data for self-medication-related behaviors before and during the lockdown showed that such behaviors appeared to decrease during the lockdown, some people who had previously self-medicated appearing not have done this during the lockdown. Analysis revealed only a few socio-demographic differences between people continuing and not continuing to exhibit self-medication-related behaviors during lockdown (see Appendix C). So, the best explanation for the apparent decrease in the behaviors at issue is likely to be the disparity in times between the periods compared (3 months vs. a person’s whole life-time). People also suffered less from common ailments (colds and flu) during lockdown, and decreases in the incidence of infectious diseases ranged between 20 and 75% in Poland [37]. 

The most interesting observation we made is that during the relatively short three-month lockdown period, in which numbers of other non-COVID-19 infectious disease cases declined, self-medication-related behaviors occurred among people who had never previously engaged in them. Our data indicated that during the lockdown as many as 15.4% of respondents refrained from consulting a doctor despite having worrying symptoms (12.1% had done this for the first time in their lives). Such behavior can be explained by fear of the coronavirus, media reports saying that people have been afraid to call ambulances, people often calling too late because they fear being infected with the virus by paramedics or in hospital [24,25].

Every fifth respondent (19.4%) had bought prescription medication just in case they might need it at some future time point, 15.5% of people doing this when they had not done so before the lockdown. Changes in the functioning of the health service and a fear of not being able to contact a doctor may have contributed to these people’s desire to protect themselves through this precautionary buying of medication. This behavior might also have been encouraged by the fact that pharmacists were allowed to issue prescriptions when patients were unable to see a doctor [38]. Media discussions raised ethical concerns about this situation, and about the possibility that pharmacy owners may be abusing this right by forcing pharmacists to prescribe large amounts of medications to patients simply because they desired them, rather than prescribing them only when a patient’s health was in danger as stated by the law [39]. As well as having an impact on pharmacists’ behavior, this unusual lockdown period and the removal of some legal restrictions on access to Rx drugs may also have led patients to assume much greater autonomy in making decisions to acquire drugs, take them, and perhaps even give other people access to them. These latter two possibilities are raised by Segall’s [40] research, which has shown that having supplies of Rx drugs at home can lead to their inappropriate use, for example, people using them to treat ailments other than those they are intended for, and sharing them with other household members.

Our research showed that during the lockdown 16.9% of respondents took Rx drugs without consulting a doctor (10.2% of people doing this when they had not previously done so), and that a similar percentage (16.6%) admitted to taking medication as a precaution against becoming ill (11.3% not previously having done so). Relatively fewer people (only 8.3% of respondents) indicated that, according to their perceptions, they had talked a doctor into prescribing a drug (7.0% not previously having done so). With respect to non-medical purposes (the enhancement of physical/cognitive performance), 17.9% of respondents took medications (10.8% not previously having done so). Here, it should be noted that the pandemic situation might have made lifestyle prescription drugs more accessible by enabling pharmacists to prescribe drugs, and by boosting the development of telemedicine channels. The latter is particularly relevant given that Fox and Ward’s [21] research showed that their respondents’ preferred method of obtaining lifestyle drugs was through online consultation because they thought it less likely that they would be denied access to their desired drug this way.

Importantly, when behaviors were aggregated, the mean values of both the general index and the lockdown index were relatively low. The general index data showed that 26.8% of Poles never engaged in behaviors related to inappropriate self-medication, and that only 9.2% of respondents engaged in all six types of behavior considered. With respect to the lockdown index, the majority of Poles (54.4%) declared that they had not engaged in any of the behaviors during the lockdown, and only 2.1% of respondents engaged in all six types of behavior. 

In the next part of our discussion we focus mainly on answering our second research question, which sought to identify the characteristics of Poles whose self-medication-related behaviors exhibited the most change during the lockdown period. Here, linear regression analyses showed that only two socio-demographic features—greater frequency of religious practices and the presence of children under 18 in a household—were predictive of higher scores on the lockdown index. 

Religion is often associated with positive health outcomes: religious people often have better health, adapt faster to health problems, and respond better to treatment [41]. In this context, the fact that the regression analysis showed that more religious people were characterized by higher lockdown index scores than less religious people may seem surprising. One tentative explanation of this result is that religiosity is strongly positively correlated with trust in information obtained from informal sources (spiritual leaders, family and friends, and websites of religious organizations), which are not necessarily reliable and may contradict scientific data [42]. Thus, this tendency might have fueled people’s faith in unverified information concerning the efficacy of various medications as prophylactics against the new coronavirus. Of course, such a thesis requires verification in future studies.

The observation that the presence of children under the age of 18 in a household predicted a greater tendency to self-medicate during lockdown might be explained by the likelihood that children’s parents have less time, a greater tendency to self-medicate in the event of small ailments perhaps resulting from visits to a doctor being considered more troublesome by parents. Also, parents may value their children’s health needs more than their own. For example, research shows that an intense need to care for their children may make it difficult for some mothers to lead a healthy lifestyle [43]. The previously mentioned time considerations may also lead people to self-medicate their children, and this can have highly negative consequences [44].

ANOVA showed that the least educated people had a greater tendency to engage in the behaviors studied than those with a secondary education (*p* < 0.05). Previous data show that Polish people with the lowest educational attainment use the most Rx drugs [45] but the least OTC drugs [28,45], although worldwide research on self-medication is inconclusive as to whether level of education can be adduced as an explanatory variable with respect to the use of such drugs. Some studies indicate that self-medication increases with level of education, this being explained, among other things, by more educated people being more certain about the correctness of their self-diagnoses, having greater autonomy, and having better knowledge of medicinal drugs [46,47]. However, other studies show that self-medication tendencies are greater in illiterate people and those with low levels of education [48,49], such observations being explained by the fact that education increases criticism and skepticism, this making people less inclined to believe that there is a pill for every condition; a proposition which sociological researchers have shown to be fostered by aggressive pharmaceutical marketing [50]. 

Interestingly no effects of gender or age were identified. While the former finding is not surprising given that the literature on self-medication is inconsistent as to whether men or women are more prone to self-medication [10], the latter finding may be considered unexpected since many studies indicate that self-medication increases with age [10]. However, the finding may be attributable to the possibility that many of the oldest (and often the least healthy) members of Polish society might have been denied the chance to participate in the study because of their tendency to not use the Internet.

To provide a better description of people scoring relatively highly on the lockdown index, we examined the responses to various individual survey items concerning fears, mental well-being, identification of the health hazard and avoiding it, behavior in the event of contracting the virus, and beliefs in controversial theories. The results showed that people who experienced various fears (about their health, work, and finances) had higher lockdown index scores, these findings being consistent with longstanding observations linking higher levels of anxiety with increased self-medication [51]. Also, higher lockdown index scores were found for people who declared that the prolonged period of social isolation had negatively affected their mental well-being (*p* < 0.001): these people may have attempted to improve their mental well-being by medicinal means. Such behaviors can only have been encouraged by the pharmaceutical industry’s tendency to promote “disease mongering” whereby (in this case) normal emotional states (e.g., mood swings and anxiety) are touted as diseases requiring pharmacological treatment—research has confirmed that Poles are subjected to such aggressive pharmaceutical marketing practices [50].

People with higher lockdown index scores also showed a greater interest in statistics relating to the pandemic (*p* < 0.001), and made greater attempts to avoid the threats it posed by engaging in preventive behaviors, for example, by ensuring they had an adequate food supply (*p* < 0.001) and avoiding going to the pharmacy (*p* < 0.001): such people’s self-medication behaviors displayed a good fit with this cautious profile. Given this, it was surprising to find that people who declared that they would not know what to do if they observed symptoms of the coronavirus in themselves or members of their household obtained higher lockdown index scores than those with such knowledge (*p* < 0.001). Furthermore, it might also be considered surprising that higher lockdown index scores were obtained by people who declared that they would not immediately contact the appropriate infectious disease hospital or sanitary department if they developed coronavirus symptoms (*p* < 0.01). This pattern of results might be explained by positing that such people did not trust the healthcare system. This thesis is strengthened by the fact that higher lockdown indices were observed for people indicating agreement with controversial statements asserting that “rather than medical drugs, more natural methods, a proper diet, or alternative medications are the best way to fight the coronavirus” (*p* < 0.05), and “pharmaceutical companies are responsible for releasing the coronavirus” (*p* < 0.01). Verification of this thesis would require further more in-depth research.

It should be acknowledged that the present study has some limitations. Foremost among these is that data were collected via online surveying of an Internet panel. In Poland, only 70% of the population declare regular use of the Internet [52], and the possibility of expressing their views was unavailable to people without access to the Internet. As mentioned earlier, such people would be expected to be disproportionately elderly, and therefore are more likely to make frequent use of medications due to chronic diseases, and be more exposed to the negative effects of developing COVID-19. It is also possible that people registering to complete surveys for prizes/money via Internet research panels may be characterized by a narrower range of attributes (e.g., in terms of skills/knowledge/financial needs) than the general population of a country. So, despite the use of a quota sample representing the socio-demographic make-up of Polish society, the data may have been somewhat distorted. Additionally, the study did not collect information about categories of medication used for self-medication (e.g., antibiotics). Nor did it consider factors motivating self-medication: such knowledge would have been highly useful in the context of the analyses performed. On the other hand, the greatest strength of the study is that it was carried out at a unique point in time—immediately after the Polish lockdown period—and people were asked about the period they had just experienced. It would be impossible to replicate this study because human memory is unreliable, and to the best of our knowledge this is the first scientific report dealing with the issue of self-medication in Poland during the lockdown period. In general, there is not much research on self-medication in Poland, and the present research enriches existing Polish patient care knowledge. The study has identified the groups that are most prone to self-medication, and should help to target public health programs at these sections of society.

## 5. Conclusions

While responsible self-medication may be beneficial in a situation such as a pandemic, inappropriate self-medication presents dangers. Our study showed that all six behaviors connected with self-medication were more prevalent before the Polish lockdown than during the lockdown, but this may have been due to the large disparity in the time periods compared (respondents’ whole lives vs. the three-month lockdown period). However, during the lockdown period, behaviors connected with self-medication occurred in people who had not previously exhibited them. People with higher lockdown index scores were characterized by greater fears (about their health, finance, and employment) and poorer mental well-being, and were more inclined to believe in controversial theories relating to the new coronavirus. When controlling other variables in multiple regression analysis, such people were also shown to be more likely to have children under 18 in their households and to be more religious. It is important to discuss the issue of self-medication toward the end of developing appropriate public health programs which can help people properly manage their medication at a time when the availability of doctors is still limited and threats of future lockdowns persist.

## Figures and Tables

**Table 1:** Percentages (and raw numbers) for responses to statements concerning self-medication behaviors before and during the lockdown period.

Please Indicate Whether the Following Situations Have Occurred	Yes, Both before and during the Lockdown% (*n*)	Yes, Only during the Lockdown % (*n*)	Yes, Only before the Lockdown % (*n*)	No, Never % (*n*)	Total
You have taken medication for the enhancement of physical/cognitive performance	7.1 (72)	10.8 (109)	18.3 (185)	63.8 (645)	100 (1011)
You have taken prescription medication ^1^ without consulting a doctor	6.7 (68)	10.2 (103)	22.4 (227)	60.5 (613)	100 (1011)
You have refrained from consulting a doctor despite having worrying symptoms	3.1 (31)	12.1 (123)	26.0 (263)	58.8 (595)	100 (1012)
You have bought prescription medication just in case you might need it ^2^	3.9 (40)	15.5 (157)	20.0 (203)	60.5 (613)	100 (1013)
You have talked a doctor into prescribing medication	1.3 (13)	7.0 (71)	11.4 (115)	80.4 (814)	100 (1013)
You have taken medication as a precaution against becoming ill	5.3 (54)	11.3 (114)	17.4 (176)	66.0 (669)	100 (1013)

Note: Differences in total sample sizes are due to single missing data points. *n*—number of respondents. ^1^ In Poland, people can only buy prescription medication when they have a prescription from a person entitled to issue prescriptions (a physician, dentist, nurse, midwife, military surgeon, or pharmacist). Which drugs each type of practitioner can prescribe, and the circumstances under which they can prescribe them, are both specified exactly in Polish law. The law also specifies which medications are Rx (and this is not always the same as in other countries), and the categories of patient which can be prescribed discounted medication or medication which is free under a reimbursement scheme. ^2.^ In Poland, it is necessary to have a prescription each time prescription drugs are obtained from a pharmacy. Multiple visits to a pharmacy using the same prescription are not permitted: a person either has to obtain a new prescription every time they require a single round of medication or they have to obtain multiple rounds of medication (e.g., for a six-month period) with a single prescription and then store all the medication at home.

**Table 2:** Results of two linear regression analyses predicting values of the general index and lockdown index.

Independent Variable	Dependent Variable									
	Analysis 1 General Index					Analysis 2 Lockdown Index				
	*B*	SE *B*	β	*t*	*p*	*B*	SE *B*	β	*t*	*p*
Age (ascending)	0.046	0.125	0.012	0.371	0.711	0.019	0.038	0.018	0.511	0.609
Population size of place of residence (ascending)	−0.016	0.051	−0.011	−0.313	0.754	0.035	0.033	0.036	1.049	0.295
Education (ascending)	0.122	0.045	0.091	2.696	**0.007**	−0.023	0.054	−0.014	−0.427	0.669
Frequency of participation in religious practices (descending)	0.002	0.074	0.001	0.032	0.975	−0.111	0.029	−0.126	−3.763	**<0.001**
Life satisfaction (descending)	−0.187	0.040	−0.154	−4.682	**<0.001**	−0.023	0.049	−0.017	−0.476	0.634
Gender (F-M)	0.064	0.066	0.034	0.978	0.328	−0.011	0.092	−0.004	−0.122	0.903
Self-assessment of health (descending)	0.315	0.088	0.132	3.589	**<0.001**	0.125	0.065	0.073	1.939	0.053
Children in household (none–at least one)	0.535	0.127	0.140	4.204	**<0.001**	0.282	0.094	0.102	3.010	**0.003**
Constant	1.326	0.459		2.889	0.004	0.972	0.338		2.874	0.004

*B*—unstandardized regression coefficient, SE—standard error, β—standardized regression coefficient, *t*—*t*-test value, *p*—significance level.

**Table 3:** Results of *t*-tests on the general and lockdown indices for groups differing in life satisfaction, the presence of children in a household, and engagement in religious practices.

Variable	General Index						Lockdown Index					
	*n*	M	SD	*t*	*p*	d	n	M	SD	*t*	*p*	d
Life satisfaction ^1^												
Satisfied	770	2.02	1.88	−2.85	**<0.01**	0.24	770	0.94	1.43	−0.61	>0.05	*n*/a
Dissatisfied	189	2.50	2.14				189	1.01	1.37			
Children in household ^2^												
None	414	1.78	1.76	−4.28	**<0.001**	0.28	414	0.78	1.20	−3.22	**<0.01**	0.20
At least one	506	2.31	1.98				506	1.06	1.49			
Religious practices												
Regular	418	2.41	2.12	4.30	**<0.001**	0.28	418	1.13	1.53	3.64	**<0.001**	0.23
Sporadic or not at all	595	1.87	1.75				595	0.80	1.28			

*n*—number of respondents, M—mean, SD—standard deviation, *t*—*t*-test value, *p*—significance level, d—Cohen’s d. ^1^ People responding “Hard to say” (*n* = 54) were excluded from this analysis. ^2^ People under 18 years of age.

**Table 4:** Results of one-way ANOVAs on the general and lockdown indices for groups differing in education, place of residence, and self-assessed general health.

Independent Variable	*n*	M	SD	F	*p*	Post-hoc Comparisons (Bonferroni Corrected)		
							Mean Difference	*p*
Education								
General Index								
Primary, lower secondary, vocational	124	2.44	2.06	4.10 (21,010)	<0.05	Secondary education	0.50	**<0.05**
						Higher education	0.24	>0.05
Secondary education	531	1.94	1.86			Primary etc.	−0.50	**<0.05**
						Higher education	−0.25	>0.05
Higher education	358	2.19	1.97			Primary etc.	−0.24	>0.05
						Secondary education	0.25	>0.05
Lockdown index								
Primary, lower secondary, vocational	124	1.18	1.60	3.33 (21,010)	<0.05	Secondary education	0.34	**<0.05**
						Higher education	0.19	>0.05
Secondary education	531	0.85	1.32			Primary etc.	−0.34	**<0.05**
						Higher education	−0.15	>0.05
Higher education	358	1.00	1.43			Primary etc.	−0.19	>0.05
						Secondary education	0.15	>0.05
Place of residence (population size)								
General index								
Village	345	2.16	2.07	3.24 (41,008)	<0.05	City of up to 19,999	0.44	>0.05
						City 20,000–199,999	0.24	>0.05
						City 200,000–499,999	0.02	>0.05
						City of over 500,000	−0.36	>0.05
City of up to 19,999	110	1.72	1.69			Village	−0.44	>0.05
						City 20,000–199,999	−0.19	>0.05
						City 200,000–499,999	−0.42	>0.05
						City of over 500,000	−0.80	**<0.05**
City 20,000–199,999	228	1.91	1.80			Village	−0.24	>0.05
						City of up to 19,999	0.19	>0.05
						City 200,000–499,999	−0.22	>0.05
						City of over 500,000	−0.60	**<0.05**
City 200,000–499,999	198	2.13	1.86			Village	−0.02	>0.05
						City of up to 19,999	0.42	>0.05
						City 20,000–199,999	0.22	>0.05
						City of over 500,000	−0.38	>0.05
City of over 500,000	132	2.51	2.00			Village	0.36	>0.05
						City of up to 19,999	0.80	**<0.05**
						City 20,000–199,999	0.60	**<0.05**
						City 200,000–499,999	0.38	>0.05
Self-assessment of health								
General index								
Very good	229	1.80	1.95	6.88 (21,009)	<0.001	Good	−0.18	>0.05
						Moderate	−0.59	**<0.01**
						Bad	−1.00	**<0.01**
Good	468	1.99	1.81			Very good	18	>0.05
						Moderate	−0.41	**<0.05**
						Bad	−0.82	**<0.05**
Moderate	263	2.40	2.05			Very good	0.59	**<0.01**
						Good	0.41	**<0.05**
						Bad	−0.41	>0.05
Bad (poor and very poor)	53	2.81	1.99			Very good	1.00	**<0.01**
						Good	0.82	**<0.05**
						Moderate	0.41	>0.05

*n*—number of respondents, M—mean, SD—standard deviation, F—ANOVA F-test value, *p*—significance level, d—Cohen’s d.

**Table 5:** Results of *t*-tests on the lockdown index for groups differing in their acceptance of various individual statements.

		*n*	M	SD	*t*	*p*	*d*
**Fear**							
I feel fear for my health	Yes ^1^	580	1.08	1.49	5.40	**<0.001**	0.37
	No ^2^	286	0.61	1.01			
I feel fear for the health and lives of my loved ones	Yes	693	0.98	1.40	2.99	**<0.01**	0.24
	No	178	0.67	1.15			
I am afraid that I will be financially broken by the prolonged pandemic	Yes	560	1.05	1.50	4.27	**<0.001**	0.29
	No	260	0.67	1.05			
I am afraid of losing my job because of the situation	Yes	434	1.18	1.61	5.36	**<0.001**	0.38
	No	321	0.66	1.05			
**Mental well-being**							
The prolonged period of social isolation is negatively affecting my mental well-being	Yes	609	1.05	1.47	4.60	**<0.001**	0.33
	No	230	0.63	1.05			
**Identifying the health hazard and avoiding it**							
I follow information about the pandemic daily, and monitor incidence statistics	Yes	516	1.09	1.51	4.16	**<0.001**	0.27
	No	342	0.71	1.33			
I have avoided going inside pharmacies since the pandemic started	Yes	313	1.27	1.62	5.12	**<0.001**	0.38
	No	488	0.73	1.16			
I have acquired appropriate food supplies to allow myself to stay at home for a long period of time	Yes	417	1.17	1.57	4.63	**<0.001**	0.32
	No	421	0.72	1.17			
In the current situation, I would not offer my hand to greet anyone except members of my household	Yes	461	1.11	1.51	3.28	**<0.001**	0.28
	No	310	0.73	1.23			
I would get vaccinated if a coronavirus vaccine was already available	Yes	452	1.05	1.47	2.95	**<0.01**	0.21
	No	305	0.76	1.25			
I believe that defrosting of the economy and lifting restrictions has been done too quickly	Yes	404	1.11	1.51	3.11	**<0.01**	0.23
	No	345	0.80	1.23			
**Behavior in the event of contracting the virus**							
If I developed coronavirus symptoms, I would immediately contact the appropriate infectious disease hospital or sanitary department	Yes	772	0.83	1.29	−3.41	**<0.01**	0.46
	No	80	1.56	1.86			
I know exactly what to do if I observe coronavirus symptoms in myself or members of my household	Yes	696	0.85	1.31	−3.95	**<0.001**	0.45
	No	108	1.55	1.75			
**Beliefs in controversial theories**							
Rather than medical drugs, more natural methods, a proper diet, or alternative medications are the best way to fight the coronavirus	Yes	258	1.22	1.67	2.60	**<0.05**	0.22
	No	390	0.89	1.33			
Pharmaceutical companies are responsible for releasing the coronavirus	Yes	177	1.37	1.82	2.98	**<0.01**	0.27
	No	495	0.93	1.35			

^1^ “Yes” constitutes responses of definitely yes and probably yes; ^2^ “No” constitutes responses of probably not and definitely not. Data for people responding “Hard to say” were removed from analyses. *n*—number of respondents, M—mean, SD—standard deviation, *t*—*t*-test value, *p*—significance level, d—Cohen’s d.

## Supplementary Materials

The following are available online at https://figshare.com/articles/dataset/Poland_-_Covid_-19_Second_Wave/13071875.

## Appendix A

**Table A1:** Participants’ socio-demographic characteristics.

Characteristic	*n*	%
Gender		
Female	544	53.7
Male	469	46.3
Age (years)		
18–29	335	33.1
30–39	263	26.0
40–49	175	17.3
50–59	141	13.9
60+	99	9.8
Education		
Primary, lower secondary, vocational	124	12.3
Secondary education	531	52.4
Higher education	358	35.3
Self-assessment of health		
Very good	229	22.6
Good	468	46.2
Moderate	263	26.0
Poor	42	4.1
Very poor	11	1.1
Place of residence (population size)		
Village	345	34.1
City of up to 19,999	110	10.9
City of 20,000–199,999	228	22.5
City of 200,000–499,999	198	19.5
City of over 500,000	132	13.0
Life satisfaction		
Satisfied	754	75.3
Dissatisfied	163	16.3
Hard to say	84	8.4
Religiosity (frequency of participation in religious practices)		
Several times a week	29	2.9
Once a week	256	25.3
1–2 times a month	133	13.1
Several times a year	254	25.1
Once every few years	87	8.6
Not at all	254	25.1
Children (people under 18 years of age) in household		
0	414	45.0
1	366	39.8
2	109	11.8
3	28	3.0
4	3	0.3
Financial situation		
Very good	78	7.7
Good	368	36.3
Moderate	465	45.9
Poor	84	8.3
Very poor	18	1.8

## Appendix B

**Table A2:** Pearson’s *r* correlation matrix for relationships between statements used to create the general index (*N* = 1013).

	You Have Taken Medication for the Enhancement of Physical/Cognitive Performance	You Have Taken Prescription Medication without Consulting a Doctor	You Have Refrained from Consulting a Doctor Despite Having Worrying Symptoms	You Have Bought Prescription Medication Just in Case You Might Need It	You Have Talked a Doctor into Prescribing Medication	You Have Taken Medication as a Precaution against Becoming ill
You have taken medication for the enhancement of physical/cognitive performance						
You have taken prescription medication without consulting a doctor	0.401 **					
You have refrained from consulting a doctor despite having worrying symptoms	0.302 **	0.379 **				
You have bought prescription medication just in case you might need it	0.334 **	0.347 **	0.260 **			
You have talked a doctor into prescribing medication	0.388 **	0.442 **	0.379 **	0.444 **		
You have taken medication as a precaution against becoming ill	0.411 **	0.332 **	0.298 **	0.355 **	0.427 **	

** *p* < 0.001 (2-tailed).

**Table A3:** Pearson’s *r* correlation matrix between statements used to create the lockdown index (*N* = 1013).

	You Have Taken Medication for the Enhancement of Physical/Cognitive Performance	You Have Taken Prescription Medication without Consulting a Doctor	You Have Refrained from Consulting a Doctor Despite Having Worrying Symptoms	You Have Bought Prescription Medication Just in Case You Might Need It	You Have Talked a Doctor into Prescribing Medication
You have taken medication for the enhancement of physical/cognitive performance					
You have taken prescription medication without consulting a doctor	0.320 **				
You have refrained from consulting a doctor despite having worrying symptoms	0.283 **	0.338 **			
You have bought prescription medication just in case you might need it	0.233 **	0.265 **	0.271 **		
You have talked a doctor into prescribing medication	0.355 **	0.352 **	0.401 **	0.314 **	
You have taken medication as a precaution against becoming ill	0.277 **	0.338 **	0.218 **	0.297 **	0.347 **

** *p* < 0.001 (2-tailed).

## Appendix C

**Table A4:** Socio-demographic distributions of self-medication-related behaviors before and during the lockdown.

Statement	Characteristic		Yes, before and during the Lockdown % (n_o_/n_e_) SR	Yes, only before the Lockdown % (n_o_/n_e_) SR	Pearson’s Chi-Square
You have taken prescription medication without consulting a doctor	Children (people under 18 years of age) in household	0	23.9 (26/24.8) 0.3	76.3 (81/84.2) -0.1	***p*****< 0.05** χ ^2^ = 9.96
	
		1	17.6 (21/27.0) −1.2	82.4 (98/92.0) 0.6	
	
		2	39.5 (15/8.6) 2.2	60.5 (23/29.4) −1.2	
	
		3	0.0 (0/1.6) −1.3	100 (7/5.4) 0.7	
	
You have bought prescription medication just in case you might need it	Life satisfaction	Satisfied	17.1 (31/29.8) 0.2	82,9 (150/151.2) −0.1	***p* < 0.01** χ ^2^ = 11.15
	
		Dissatisfied	7.7 (4/8.6) 1.6	92.3 (48/43.4) 0.7	
	
		Hard to say	50.0 (5/1.6) 2.6	50 (5/8.4) −1.2	
	
	Self-assessment of health	Very good and good	16.3% (36/36.3) −0.1	83.8% (134/133.8) 0.0	***p* < 0.05** χ^2^ = 6.15
	
		Moderate	11.9 (8/11.0) −0.9	88.1 (59/56.0) 0.4	
	
		Poor and very poor	37.5 (6/2.6) 2.1	62.5 (10/13.4) −0.9	
	
	Religiosity (frequency of participation in religious practices)	Several times a week	14.3 (1/1.2) −0.1	85.7 (6/5.8) 0.1	***p* < 0.05** χ ^2^ = 15.11
	
		Once a week	9.9 (6/10.2) −1.3	90.3 (56/51.8) 0.6	
	
		1–2 times a month	11.6 (5/7.1) −0.8	88.4 (38/35.9) 0.3	
	
		Several times a year	13.6 (9/10.9) −0.6	86.4 (57/55.1) 0.3	
	
		Once every few years	44.4 (8/3) 2.9	55.6 (10) −1.3	
	
		Not at all	23.4 (11) 1.2	76.6 (36) −0.5	
	
You have taken medication as a precaution against becoming ill	Gender	Female	29.2 (33/26.5) 1.3	70.8 (80/86.5) −0.7	***p* < 0.05** χ^2^ = 4.05
	
		Male	17.9 (21/27.5) −1.2	82.1 (96/89.5) 0.7	
	
	Age	18–29	26.2 (22/19.7) 0.5	73.8 (62/64.3) −0.3	***p* < 0.05** χ ^2^ = 4.05
	
		30–39	16.1 (10/14.6) −1.2	83.9 (52/47.4) 0.7	
			−		
		40–49	33.3 (14/9.9) 1.3	66.7 (28/32.1) −0.7	
	
		50–59	29.6 (8/6.3) 0.7	70.4 (19/20.7) −0.4	
	
		60+	0.0 (0/3.5) −1.9	100 (15/11.5) 1.0	
	
	Life satisfaction	Satisfied	28.1 (48/40.1) 1.2	71.9 (123/130.9) −0.7	***p* < 0.05** χ^2^ = 7.82
	
		Dissatisfied	10.2 (5/11.5) −1.9	89.8 (44) 1.1	
		
		Hard to say	10 (1/2.3) −0.9	90.0 (9/7.7) 0.5	
		

no—observed count, ne—expected count, SR—standard residuals. Note: The table only includes results for socio-demographic features which had significant associations with self-medication-related behaviors.
